# Measurement of endotracheal tube secretions volume by micro computed tomography (MicroCT) scan: an experimental and clinical study

**DOI:** 10.1186/1471-2253-14-22

**Published:** 2014-03-28

**Authors:** Andrea Coppadoro, Giacomo Bellani, Alfio Bronco, Roberto Borsa, Alberto Lucchini, Simone Bramati, Leonello Avalli, Roberto Marcolin, Antonio Pesenti

**Affiliations:** 1 Department of Health Sciences, University of Milan-Bicocca, Monza, Italy; 2 Department of Anesthesia and Intensive Care, San Gerardo Hospital, Monza, Italy; 3 Department of Clinical Pathology, Unit of Microbiology, San Gerardo Hospital, Monza, Italy

**Keywords:** Endotracheal tube, Micro computed tomography, Secretions, Volume, Cross-sectional area

## Abstract

**Background:**

Biofilm accumulates within the endotracheal tube (ETT) early after intubation. Contaminated secretions in the ETT are associated with increased risk for microbial dissemination in the distal airways and increased resistance to airflow. We evaluated the effectiveness of micro computed tomography (MicroCT) for the quantification of ETT inner volume reduction in critically ill patients.

**Methods:**

We injected a known amount of gel into unused ETT to simulate secretions. We calculated the volume of gel analyzing MicroCT scans for a length of 20 cm. We then collected eleven ETTs after extubation of critically ill patients, recording clinical and demographical data. We assessed the amount of secretions by MicroCT and obtained ETT microbiological cultures.

**Results:**

Gel volumes assessed by MicroCT strongly correlated with injected gel volumes (p < 0.001, r^2^ = 0.999).

MicroCT revealed the accumulation of secretions on all the ETTs (median 0.154, IQR:0.02-0.837 mL), corresponding to an average cross-sectional area reduction of 1.7%. The amount of secretions inversely correlated with patients’ age (p = 0.011, rho = −0.727) but not with days of intubation, SAPS2, PaO_2_/FiO_2_ assessed on admission. Accumulation of secretions was higher in the cuff region (p = 0.003). Microbial growth occurred in cultures from 9/11 ETTs, and did not correlate with secretions amount. In 7/11 cases the same microbes were identified also in tracheal aspirates.

**Conclusions:**

MicroCT appears as a feasible and precise technique to measure volume of secretions within ETTs after extubation. In patients, secretions tend to accumulate in the cuff region, with high variability among patients.

## Background

The endotracheal tube (ETT) allows physicians to maintain adequate alveolar ventilation despite patient’s respiratory muscles failure and loss of airway defense. However, the presence of an ETT is associated with infectious and non-infectious complications [1,2]. The ETT involvement in the development of airway bacterial colonization has been extensively described, to the extent that some authors proposed to change the term ventilator-associated pneumonia in ETT- associated pneumonia [3]. Secretions cannot be spontaneously removed from the airways as happens in healthy subjects, and soon after intubation the ETT is colonized by a biofilm of extracellular polymeric substances where microbial aggregates proliferate [4,5]. Bacterial aggregates may detach from the biofilm, particularly in the advanced stage, and may be projected by the airflow into the distal airways leading to pneumonia [6,7]. With time, secretions accumulate and the inner lumen of the ETT tends to reduce its diameter, resulting in patient’s increased work of breathing [8]. Frequent ETT suctioning is part of the current clinical practice to maintain lumen patency and avoid occlusion due to mucus plugs, together with heating and humidification of inspired gases and other measures aimed at preventing the formation of biofilm [9]. However, standard care is not effective to preserve the inner lumen patency, and specific devices has been designed with the purpose of removing secretions from the ETT lumen, resolving sudden ETT occlusions and possibly reducing the risk of airways colonization and work of breathing due to increased ETT airflow resistance [10,11].

Although the relationship between biofilm and risk of pneumonia is largely described in the literature, no gold standard technique is currently available to measure biofilm amount on the ETT inner surface. Scanning electron microscopy (SEM) has been used to demonstrate the presence of a biofilm layer, substantially reduced by the use of an ETT cleaning device [12]. SEM is very sensitive, but requires a complex sample preparation and provides data on a small part of the ETT, while the total amount of biofilm within the ETT remains unknown. Acoustic reflectometry, an indirect technique based on reflections of acoustic waveforms by the ETT surface, has also been employed to demonstrate the narrower size of ETT used in patients as compared to unused ones [13].

We hypothesized that the amount of secretions present on the inner lumen of ETTs from critically ill patients could be assessed by micro computerized tomography (MicroCT) scan after extubation [14]. MicroCT scanners are designed to reach image definitions up to the micrometric scale, on much smaller fields of view than those used in the clinical practice. We report the efficacy of MicroCT in quantifying the volume of a model of secretions in a bench study. Then, we measured the volume of secretions in a set of ETT used in critically ill patients (ex-vivo), investigating possible associations between the reduction of internal ETT diameters and patient’s related factors or ETT microbial colonization.

## Methods

The local institutional review board (San Gerardo Hospital Ethical Committee) was notified in regard of the study and waived the acquisition of written informed consent, as per local regulation.

### Bench study

We injected five different aliquots (0.25 to 1.250 mL) of an ultrasound water-based polymeric gel (density 1.03 g/mL, Gima S.P.A., Gessate, Italy) by a syringe into ETTs (Kendall Curity, Covidien, Mansfield, MA) of three sizes (6.5, 7.5 and 8.0 mm of internal diameter) at 2 cm above the tip side, to simulate the presence of secretions. The gel was then distributed for 7 cm towards the vent side by compressed air; ETTs were kept horizontal during all the procedure and were then sealed to minimize gel modifications during MicroCT scan. The exact amount of injected gel was verified by pre- and post-injection ETT weighing with a high precision scale (Precisa XB220A, Precisa Gravimetrics AG, Dietikon, Switzerland). The resulting increase of ETT resistance to airflow is described in the Additional file 1.

ETT image acquisitions were performed using a MicroCT scanner (SkyScan 1176, Bruker, Belgium) at a resolution of 35 μM. The ETTs were scanned for a length of 20 cm (from approximately 1 cm above the rib-eye up to 21 cm from the tip). We analyzed CT-reconstructed images to measure the volume of injected gel, using densitometric criteria, distinguishing three main entities of increasing density: air, gel and ETT wall. The MicroCT-embedded software (CT Analyzer, Bruker, Belgium) was used for imaging-analysis. First, we selected voxels representing the water-based polymeric gel with a density close to 0 Hounsfield Units (HU). Then, we applied a filtering algorithm to remove image noise, calculating the total gel volume by 3d-analysis of the selected ETT portion.

### Observational ex-vivo study

Eleven ETTs were collected after extubation of patients admitted to an intensive care unit (ICU) at the San Gerardo Hospital, Monza, Italy. Inclusion criteria for ETT collection were: patient’s age >18 years old; intubation for more than 72 hours; use of a closed airway suction system. Nurse staff performed ETT suctioning only when considered necessary; heated humidifiers were used in all patients. At extubation, we collected the ETTs and sealed at the extremities to prevent drying of secretions. Within 24 hours we performed MicroCT scan of the ETTs, and measured secretions volumes as described above, dividing the ETTs in three parts: proximal, or ventilator side; central; and distal, or tip side. After CT scan, we performed lavages of the ETT inner surface injecting 5 ml of sterile saline solution, and the collected fluids were cultured by standard microbiological tests. Swabs of the internal ETT surface were also collected. We conducted all the procedures relative to microbiological examinations in a sterile environment to avoid external microbial contamination.

We recorded patients’ clinical (admission PaO_2_/FiO_2_ ratio, days of ICU stay, ventilator free days in the first 28 days, data for calculation of Simplified Acute Physiology Score II [SAPS2] [15]) and demographical data (sex, age). The results of the tracheal aspirate cultures closest to extubation were also recorded, if present. To assess concordance between lavages, swabs and aspirates the most relevant isolated microbe was considered, defined as the single microbe isolated with the highest growth index.

### Statistical methods

Continuous variables are reported as mean ± standard deviation unless otherwise indicated. Normality of variables’ distribution was evaluated by the Shapiro-Wilk test. The correlation coefficient between normally distributed or non-normally distributed variables was calculated using Pearson’s and Spearman’s correlation coefficients, respectively. The evaluation of resistance to airflow was performed by a two-way ANOVA for repeated measures, considering gel amount and airflow as within-subjects factors. Secretions distribution in the three ETT portions (ventilator side, central and tip side) was evaluated by one-way ANOVA, using Tukey’s post-hoc tests. Statistical analyses were performed using SPSS software version 18.0 (SPSS Inc., Chicago, IL). Statistical significance was reached when p < 0.05.

## Results

### Bench results

**Figure 1:**
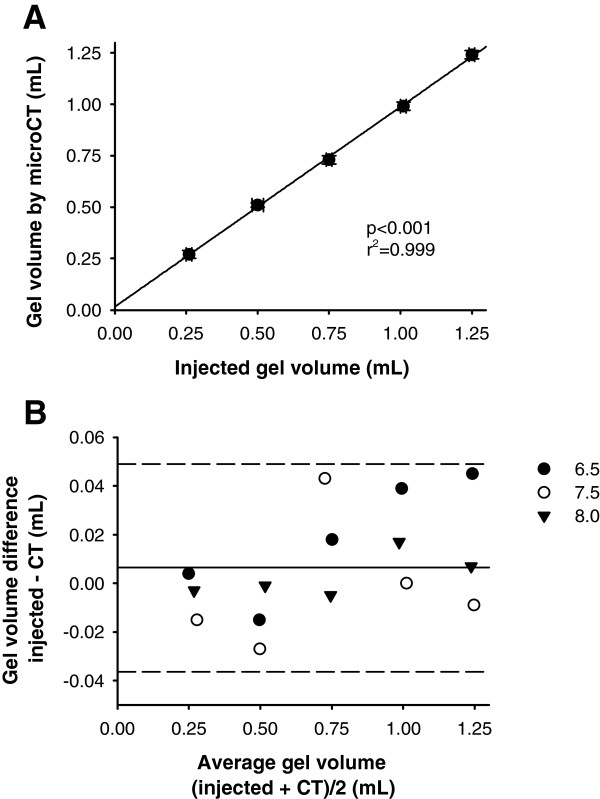
**MicroCT scan precision and accuracy in bench tests simulating the presence of secretions within ETTs.** Correlation between the amount of injected gel, simulating the secretions present at extubation, and the volume of gel measured by MicroCT scan (panel **A**). The Bland-Altman plot shows good precision in MicroCT scan assessment of volume of injected gel (average difference 0.006, 95%CI −0.036 to 0.049 mL, panel **B**). Symbols represent different ETT sizes (mm of internal diameter).

MicroCT scans showed good reliability in the assessment of the volume of our model of secretions. The amount of gel injected into unused ETTs strongly correlated with the volume assessed by MicroCT scan (p < 0.001, r^2^ = 0.999, Figure 1 panel A). The Bland-Altman plot [16] depicts the small difference between the volumes measured by two techniques (corresponding to an average difference of 0.2 ± 3.2%) and the narrow 95% confidence intervals of the differences (−0.036 to 0.049 mL, Figure 1 panel B, corresponding to a 95%CI of −6.1 to 6.5%). The effects of gel injection on resistance to airflow are reported in the Additional file 2: Figure E1.

### Ex vivo microCT

**Figure 2:**
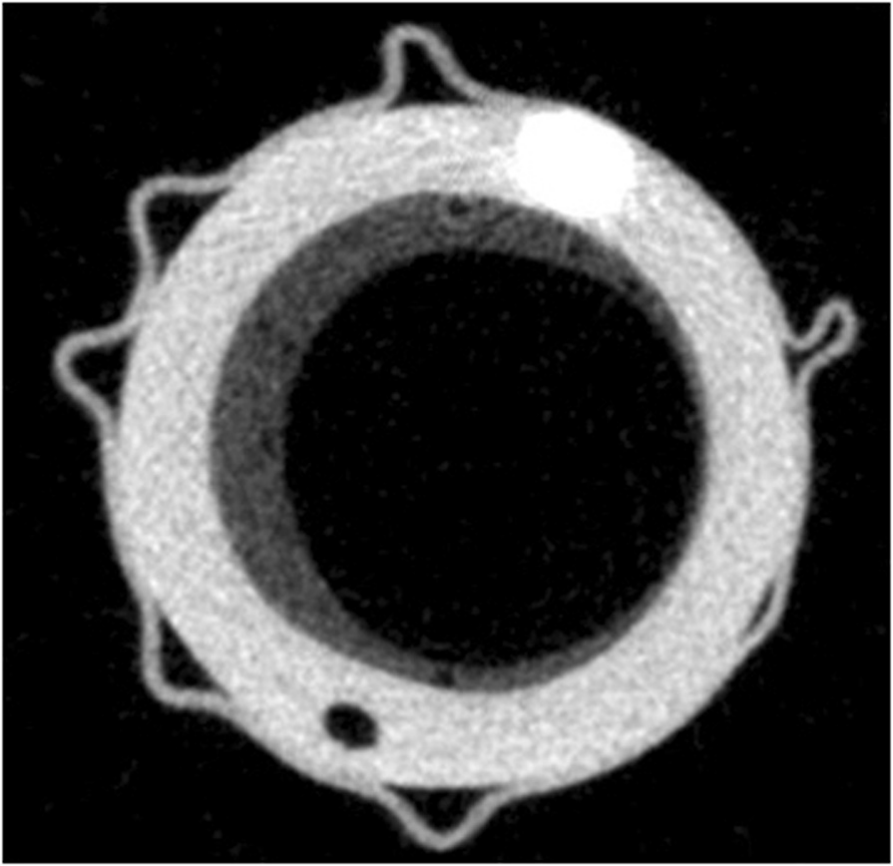
**Exemplary ETT section obtained by MicroCT scan.** Representative image obtained by MicroCT scan of an ETT collected after 10 days of intubation. The abundant secretions layer (dark grey,) is adherent to the plastic material of the ETT (light grey) in the area below the cuff.

Eleven ETTs collected after patients’ extubation were analyzed; 9 patients were successfully weaned, 2 were extubated post mortem. Patients’ demographic and clinical characteristics are reported in the Additional file 3: Table E1. MicroCT scan (Figure 2 and Figure E2, Additional file 4) revealed the presence of secretions in all the 11 studied ETTs, ranging from 0.007 to 1.103 mL (median 0.154, IQR: 0.02 to 0.837 mL) and corresponding to a median cross-sectional area reduction of 1.7% (IQR: 0.2 to 8.3%).

**Figure 3:**
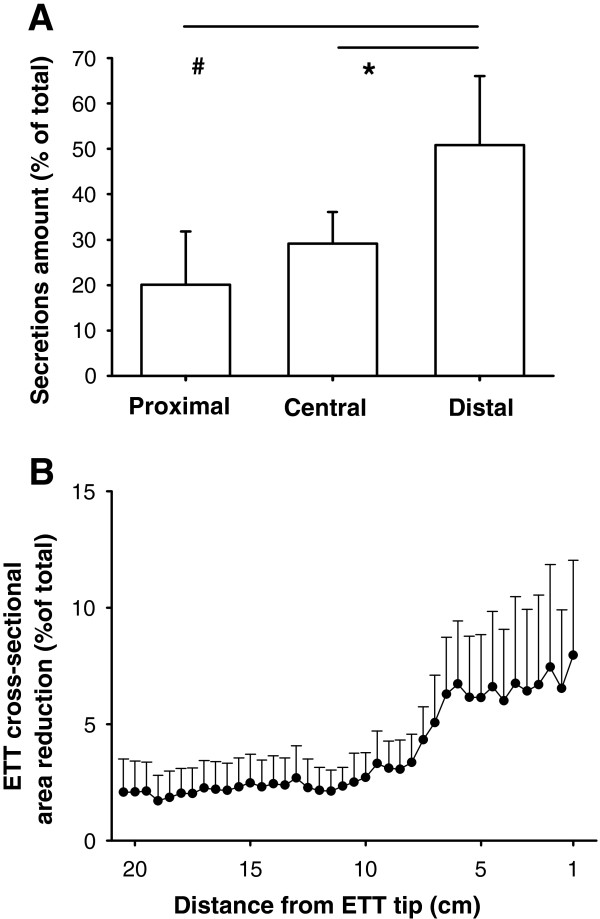
**Distribution of secretions within the ETT.** Secretions volume distribution within the 11 studied ETTs is not homogeneous (panel **A**), resulting higher in the distal portion (tip side). Correspondingly, the average cross-sectional area reduction tends to increase close to the ETT tip (panel **B**).

The volume of secretions was inversely related to patients’ age (Spearman’s rho = −0.727, p = 0.011, Additional file 5: Figure E3), while it was not associated with ICU stay, 28-day-ventilator free days, SAPS2, PaO_2_/FiO_2_ ratio at ICU admission (all p > 0.05). Secretions distribution was not homogeneous along the ETT, with higher amounts in the distal as compared to the central and the proximal regions (p = 0.003 by ANOVA, 0.037 and 0.003 respectively, Figure 3 panel A). The average ETT cross-sectional area reduction in the distal portion was nearly 6% as compared to nominal (Figure 3, panel B). The average density of secretions was 108 ± 67 HU. Average density was associated with secretions volumes (Spearman’s rho = 0.709, p = 0.015) in the eleven studied ETTs.

### Microbiological tests

Microbiological main results are reported in Table 1. Among the 11 lavage fluids, microbial growth occurred in 9 (82%) with a median viable count of 10^3^ colony forming units (IQR: 10^2^-10^6^). ETT lavage fluid viable counts and secretions amount or density were not associated in the studied samples. *Candida spp.* (mainly C. Albicans) were the most common isolated microbes (6/11, 55%). ETTs colonized by *Candida spp.* showed a more homogeneous distribution of secretions, while the ones where *Candida spp.* were not isolated showed a higher accumulation of secretions in the distal third (35 ± 21 vs. 69 ± 16% of secretions in the distal third, respectively, p = 0.016).

**Table 1:** Microbiological testing – main results

**Length of intubation (Days)**	**ETT lavage fluid cultures**	**Viable cell count (Log CFU)**	**ETT swab cultures**	**Tracheal aspirate cultures**
**2**	No growth	0	*S. Aureus* MS	-
**22**	*Aspergillus spp.*	3	*Aspergillus spp.*	*Aspergillus spp.*
				*E. Faecium*
**21**	*C. Albicans*	3	*C. Albicans*	*C. Albicans*
**3**	*C. Albicans*	4	*C. Albicans*	*C. Albicans*
**7**	*E. Coli* ESBL	7	*E. Coli* ESBL	-
	*C. Albicans*	2	*C. Albicans*	
			*S. Aureus* MS	
**4**	*C. Albicans*	1	*C. Albicans*	No growth
**15**	*C. Tropicalis*	3	*C. Tropicalis*	*C. Tropicalis*
	Oral mixed flora	>7		Oral mixed flora
**14**	No growth	0	No growth	No growth
**10**	Oral mixed flora	6	Oral mixed flora	Oral mixed flora
**6**	*B. Catharralis* BL+	6	Oral mixed flora	Oral mixed flora
	Oral mixed flora	>7		
**7**	*S. Aureus* MR	2	*S. Aureus* MR	*S. Aureus* MR
	*C. Albicans*	2	*K. Pneumoniae*	*C. Albicans*
	*K. Pneumoniae*	2		

ETT: endotracheal tube; CFU: Colony-forming units; MS: methicillin sensitive; ESBL: extended-spectrum beta-lactamases producer; BL+: beta-lactamases producer; MR: methicillin resistant.

The most relevant isolated microbes in the ETT lavage fluids were also isolated by inner lumen swabs in 8/11 cases (72%), showing a good concordance between the two specimen collecting methods. Tracheal aspirates were available for nine patients and were collected for clinical reasons at a median of 2 days (IQR: 1 to 2 days) before extubation. Again, ETT lavage fluids and tracheal aspirates showed similar results: in 7 cases (78%) the most relevant pathogen grew in both samples. Bacterial growth in tracheal aspirates was not associated with increased volume of secretions within the ETT.

## Discussion

In this study, we investigated the possibility of using MicroCT to quantify the amount of secretions present on the inner lumen of ETTs. In bench tests, we measured the volume of a model of secretions with good precision. The volume of secretions in ETTs collected after patients’ extubation showed a high variability among samples. Secretions volume was associated with its density and with patient’s age.

The presence of biofilm within the ETT is a relevant issue. Sudden obstruction may occur, and the reduction of the internal ETT diameter results in increased resistance to airflow leading to increased patient’s inspiratory effort and possibly delayed extubation [17,18]. Acoustic reflectometry, a technique based on sound emission and reflected waves analysis, has been used since several years for the measurement of the volume of secretions covering the inner ETT surface [8,13,17]. Reflectometry is a technique developed to assess the diameter of cylindrical structures which can be performed non-invasively in spontaneous breathing patients. However, data resulting from this technique are based on an indirect estimation of the area available to airflow, and do not provide the spatial distribution and actual amount of the secretions within the ETT. In this study, we used a MicroCT scanner to perform a direct measurement of secretions volume. Data obtained in the bench evaluation were reassuring regarding the precision of the measurements, as shown by the narrow 95% confidence intervals depicted in the Bland-Altman plot [16].

Some limitations apply also to MicroCT scan. As happens in the images obtained in the clinical setting, some artifacts can appear after reconstruction. However, the analysis software allowed us to effectively remove such artifacts and obtain good-quality images. Another limitation of the technique is that it cannot be applied in vivo. Although we did not test it, in-vivo images obtained with a standard CT scanner are not definite enough to provide a reliable assessment of the ETT secretions volumes, and a dedicated instrument where only the ETT fits is required. Therefore, is not currently possible to follow the evolution of the biofilm during ICU stay by CT scan. Another limitation is that the resolution is not high enough to obtain images where the different cellular types can be distinguished (as it is instead the case with SEM); thus, the different stages of biofilm cannot be studied by MicroCT. Although the scanner we used can reach higher resolutions (up to 9 μM pixel-sixe), we decided to use the 35 μM pixel-size resolution as a balance between image quality and need of resources (time, digital storage space, computation power).

By the analyses of ETT collected from critically ill patients, we obtained a precise description of the amount, distribution and density of the secretions present along a wide portion of the ETT. The amount of secretions at extubation showed a high variability among ICU patients: 3 of the analyzed ETTs showed a secretions volume higher than 0.75 mL, while in other ETTs the secretions detected by MicroCT scan were nearly absent. The average cross-sectional area reduction (1.7%) was relatively smaller than what has been described by other authors using acoustic reflectometry (about 10% cross-sectional area reduction). Indeed, the three patients with a significant amount of secretions showed an average 10% cross-sectional area reduction, reaching levels up to 30% for several centimeters in the distal region. Changes in standard airways care over the years might help to explain differences with literature data published in the past. Moreover, we could not demonstrate any correlation between secretions amount and length of intubation or markers of patient’s severity. It is well known that biofilm amount increases with time, since current airways management practice is not effective in completely removing secretions from the ETT inner surface [19]. However, length of intubation *per se* is not associated to secretions amount, as shown in the present study and by others [8].

Taken together, all these findings suggest that some ETTs are burdened by a high amount of secretions and some other are not. Patient’s related factors, such as previous or present diseases, ability to move lung secretion towards the glottis, use of muscle relaxants may be relevant factors that influence ETT secretions accumulation, and larger studies are needed to investigate possible risk factors. In the studied population, the volume of secretions inversely correlated with patient’s age. This finding need to be confirmed in a larger cohort of patients, and might be explained by the higher capacity of younger patients to move secretions towards the ETT resulting from their preserved muscular activity, or by a possible reduced mucus production of elderly patients.

The average secretions density resulted similar to soft tissues (about 100HU). Such a density is in line with airway biofilm composition, where mucosal secretions, leukocytes and red blood cells are often present [5,20]. In the studied population, secretions volume and density were also associated, possibly because dense secretions tend to accumulate and form a biofilm which is not easily removed by standard ETT suctioning, while labile secretions might be easily removed. However, increase of biofilm density might also occur after its deposition, due to microbial activity which excrete a complex extracellular matrix [21]. Further studies are needed to confirm this finding and investigate possible underlying mechanisms.

Regarding secretions spatial distribution, we showed that the ETT subglottic area is the most affected by secretions accumulation. In a small percentage of patients, such a phenomenon is clinically relevant, with degrees of obstruction above 30%. ETT intrinsic resistance to airflow and the influence of gravity, resulting in collection of secretions in the ETT most dependent part, might be factors related to this finding [22]. If confirmed, this finding demonstrates that an ETT that appears clean in its visible part (proximal) might be extensively covered by secretions in the distal one. Interestingly, in our small cohort of patients the phenomenon was less evident in the ETTs where *Candida spp.* could be isolated. Future studies might investigate if the presence of *Candida* favors the adhesion of secretions on the ETT inner surface counteracting the effects of gravity, as suggested by the complex interactions between fungi and biofilm described in the literature [23,24]. Data from our population is limited and this preliminary observation needs to be confirmed in other studies with larger sample sizes.

Considering microbiological examinations, the majority of the collected specimens showed some form of growth; however, we could not demonstrate any association between microbial growth and secretions amount or density. Our data suggest that microbial colonization can be present irrespective of the amount of secretions, as detected by MicroCT, and pathogens might dwell even in a small layer of biofilm. This finding suggests that the resolution of MicroCT is not adequate to detect such small structures and to identify the presence of a biofilm, while other techniques (such a as SEM) are more effective to detect the presence of microbes and the complex interactions occurring within the ETT lumen biofilm.

We detected similar ETT colonizing microbes in ETT lavage fluids and tracheal aspirates requested for clinical reasons a few days before extubation, as demonstrated by others [7,25]. In our unit, tracheal aspirates are collected in a sterile manner, but without the use of protected tip catheters. Therefore, our data do not allow us to understand if a continuum of colonization is present from the ETT to the trachea, or if contamination with ETT biofilm bacteria occurs at the moment of tracheal aspirate collection. The same microbes were also collected by ETT surface swabs except in two cases, when MSSA strains were present in swab cultures but not in lavage fluids. Our data is not large enough to elucidate if a different population of microbes is present in the lower biofilm layers, collected by swabs but not by saline lavages. Whether the presence of these microbes might represent a clinically relevant factor or not, remains to be demonstrated.

## Conclusions

MicroCT scan seems to be a feasible and accurate technique to assess the amount and the distribution of secretions present within the ETT after extubation. MicroCT might be useful to understand the factors related to biofilm accumulation within the ETT of critically ill patients, and to analyze the effectiveness of measures or devices specifically employed to reduce biofilm formation. Only few ETTs from a cohort of 11 patients were burdened by a relevant amount of secretions, which tended to gather in the most dependent (subglottic) area, most likely due to the effect of gravity in a semi-recumbent patient. The volume of secretions present within the ETTs was not associated with ETT microbial colonization.

## Abbreviations

ETT: Endotracheal tube; HU: Hounsfield units; ICU: Intensive care unit; IQR: Inter-quartile range; MicroCT: Micro computed tomography; SAPS2: Simplified acute physiology score II; SEM: Scanning electron microscopy.

## Competing interests

The authors declare that they have no competing interests.

## Authors’ contributions

Conception and design: AC, GB, AP; data acquisition: AC, AB, RB, AL, SB, LA, RM; analysis and interpretation of data: AC, GB, SB, AP; manuscript drafting: AC, GB; article critical revision for important intellectual content: AC, GB, AB, RB, AL, SB, LA, RM, AP. All authors read and approved the final version of the manuscript. The presented research was funded by institutional funds from the Department of Health Sciences, University of Milan-Bicocca, Monza, Italy.

## Authors’ information

The present work has been performed in the General and Cardiac Intensive Care Units of the university-affiliated San Gerardo Hospital, University of Milan-Bicocca, Monza, Italy.

## Pre-publication history

The pre-publication history for this paper can be accessed here:

http://www.biomedcentral.com/1471-2253/14/22/prepub

## Supplementary Material

Additional file 1 **Is an Acrobat file containing the electronic supplementary materials for the** Methods **and** Results **sections of present study.** Click here for file

Additional file 2 Is an Acrobat file containing Figure E1 (Increase in resistance to airflow in a 7.5 mm ETT by increase of injected gel amount). Click here for file

Additional file 3 Is an Acrobat file containing Table E1 (Patient’s demographics and clinical characteristics). Click here for file

Additional file 4 Is an Acrobat file containing Figure E2 (Exemplary 3d-reconstruction of MicroCT scan of an ETT portion). Click here for file

Additional file 5 Is an Acrobat file containing Figure E3 (Association between patient’s age and secretion amount present within the ETT). Click here for file
