# Supplementary material for: Measurement of endotracheal tube secretions volume by micro computed tomography (MicroCT) scan: an experimental and clinical study
Source: BMC Anesthesiol. 2014 Mar 28;14:22. doi: 10.1186/1471-2253-14-22 (PMC3986655; doi:10.1186/1471-2253-14-22)
Supplement: Additional file 1 — Is an Acrobat file containing the electronic supplementary materials for the Methods and Results sections of present study. [file 1471-2253-14-22-S1.pdf]

Electronic supplementary materials

Measurement of endotracheal tube secretions volume by micro computed tomography (MicroCT) scan: an experimental and clinical study.

Andrea Coppadoro[1]; Giacomo Bellani[1,2]; Alfio Bronco[1]; Roberto Borsa[1]; Alberto Lucchini[2]; Simone Bramati[3]; Leonello Avalli[2]; Roberto Marcolin[2]; Antonio Pesenti[1,2]

[1] Department of Health Sciences, University of Milan-Bicocca, Monza, Italy

[2] Department of Anesthesia and Intensive Care, San Gerardo Hospital, Monza, Italy

[3] Department of Clinical Pathology, Unit of Microbiology, San Gerardo Hospital, Monza, Italy

Methods

The same five gel aliquots were used to assess airway resistances to airflow in a 7.5 mm ETT. We connected flow and pressure transducers to an acquisition system (Respironics CO2SMO plus!, Philips Healthcare, Andover, MA) calculating airway resistances at 30, 45 and 60 L/min of airflow by measurement of pressure drop across the ETTs. Three measurements were obtained for each combination of flow and gel aliquots.

Results

We then evaluated the impact of our model of secretions on resistance to airflow in a 7.5mm ETT, resembling quiet assisted breathing (30 L/min), controlled mechanical ventilation (45 L/min) and increased respiratory effort during assisted breathing (60 L/min). As expected, resistances increased by increasing the amount of injected gel and airflow ($p<0.001$ for both, Figure E1). The resistance increase was more pronounced at higher airflows ($p<0.001$ for interaction), i.e. doubled at 30 L/min and increased almost three times at 60 L/min of airflow after injection of 1 ml of gel.
