# Supplementary material for: Measurement of endotracheal tube secretions volume by micro computed tomography (MicroCT) scan: an experimental and clinical study
Source: BMC Anesthesiol. 2014 Mar 28;14:22. doi: 10.1186/1471-2253-14-22 (PMC3986655; doi:10.1186/1471-2253-14-22)
Supplement: Additional file 2 — Is an Acrobat file containing Figure E1 (Increase in resistance to airflow in a 7.5 mm ETT by increase of injected gel amount). [file 1471-2253-14-22-S2.pdf]

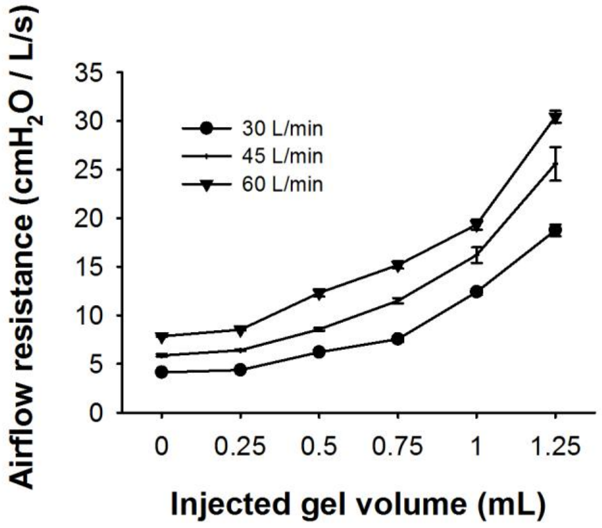

Figure E1 Increase in resistance to airflow in a 7.5 mm ETT by increase of injected gel amount, resembling the biofilm present at extubation. The increase of resistances is more pronounced at higher flows ($p<0.001$)
