# Supplementary material for: Measurement of endotracheal tube secretions volume by micro computed tomography (MicroCT) scan: an experimental and clinical study
Source: BMC Anesthesiol. 2014 Mar 28;14:22. doi: 10.1186/1471-2253-14-22 (PMC3986655; doi:10.1186/1471-2253-14-22)
Supplement: Additional file 3 — Is an Acrobat file containing Table E1 (Patient’s demographics and clinical characteristics). [file 1471-2253-14-22-S3.pdf]

Table E1. Patient's demographics and clinical characteristics

Females	Age	ICU	SAPS 2	Admission PaO_2/FiO_2	Survivors
1/11	70 ±13	8 Cardiac<br>3 General	46 ±11	214 ±54	9/11
