# Supplementary material for: Measurement of endotracheal tube secretions volume by micro computed tomography (MicroCT) scan: an experimental and clinical study
Source: BMC Anesthesiol. 2014 Mar 28;14:22. doi: 10.1186/1471-2253-14-22 (PMC3986655; doi:10.1186/1471-2253-14-22)
Supplement: Additional file 4 — Is an Acrobat file containing Figure E2 (Exemplary 3d-reconstruction of MicroCT scan of an ETT portion). [file 1471-2253-14-22-S4.pdf]

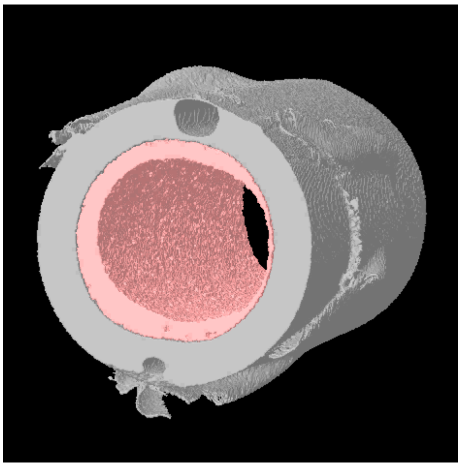

Figure E2 Exemplary 3d-reconstruction of MicroCT scan of an ETT portion. The layer of secretions present on the inner lumen in the cuff portion is highlighted in light red.
