# Supplementary material for: Measurement of endotracheal tube secretions volume by micro computed tomography (MicroCT) scan: an experimental and clinical study
Source: BMC Anesthesiol. 2014 Mar 28;14:22. doi: 10.1186/1471-2253-14-22 (PMC3986655; doi:10.1186/1471-2253-14-22)
Supplement: Additional file 5 — Is an Acrobat file containing Figure E3 (Association between patient’s age and secretion amount present within the ETT). [file 1471-2253-14-22-S5.pdf]

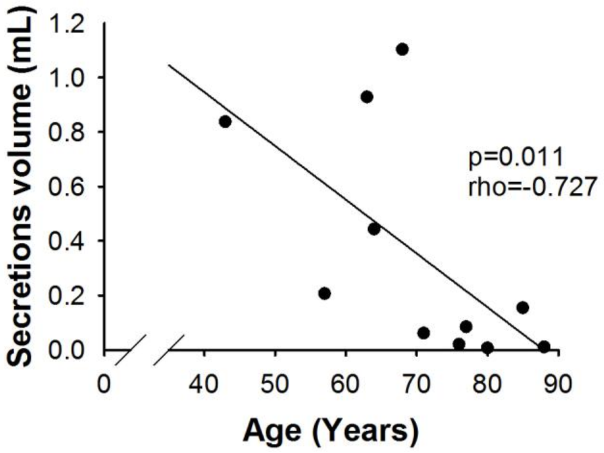

Figure E3 Association between patient's age and secretions volume present within the ETT. The highest secretion amounts were measured in ETTs of younger patients.
